# Systematic review of the performance evaluation of clinicians with or without the aid of machine learning clinical decision support system

**DOI:** 10.1007/s12553-023-00763-1

**Published:** 2023-06-13

**Authors:** Mikko Nuutinen, Riikka-Leena Leskelä

**Affiliations:** 1 Nordic Healthcare Group, Helsinki, Finland; 2 Haartman Institute, University of Helsinki, Helsinki, Finland

**Keywords:** Clinical decision support systems, Experimental design, Machine learning, Literature review

## Abstract

**Background:**

For the adoption of machine learning clinical decision support systems (ML-CDSS) it is critical to understand the performance aid of the ML-CDSS. However, it is not trivial, how the performance aid should be evaluated. To design reliable performance evaluation study, both the knowledge from the practical framework of experimental study design and the understanding of domain specific design factors are required.

**Objective:**

The aim of this review study was to form a practical framework and identify key design factors for experimental design in evaluating the performance of clinicians with or without the aid of ML-CDSS.

**Methods:**

The study was based on published ML-CDSS performance evaluation studies. We systematically searched articles published between January 2016 and December 2022. From the articles we collected a set of design factors. Only the articles comparing the performance of clinicians with or without the aid of ML-CDSS using experimental study methods were considered.

**Results:**

The identified key design factors for the practical framework of ML-CDSS experimental study design were performance measures, user interface, ground truth data and the selection of samples and participants. In addition, we identified the importance of randomization, crossover design and training and practice rounds. Previous studies had shortcomings in the rationale and documentation of choices regarding the number of participants and the duration of the experiment.

**Conclusion:**

The design factors of ML-CDSS experimental study are interdependent and all factors must be considered in individual choices.

**Supplementary Information:**

The online version contains supplementary material available at 10.1007/s12553-023-00763-1.

## Introduction

A clinical Decision Support System (CDSS) is a software device that supports clinicians in decision making. For example, a CDSS can indicate areas on an X-ray image from where a fracture can be found [1, 2], guide an endoscopist to execute examination comprehensively [3] or warn clinicians of hypoxaemia/hypotension risk [4, 5].

Recent CDSSs utilize advanced machine learning (ML) techniques. However, traditional accuracy measurements of ML algorithms are not sufficient to show that the ML-CDSS is effective also in a real clinical environment. The human decision-making process is complex and biased. It cannot be assumed that clinicians will always closely follow the recommendations of ML models [6, 7]. For that reason, it is especially important to measure the performance of ML-CDSS software being developed and to validate the functionality well in advance by using suitable experimental methods before large-scale and expensive implementation.

In this study, we reviewed recent studies in which the performance of ML-CDSSs were measured using experimental study methods. The objective was to review how experimental studies measuring the performance of clinicians with or without the aid of ML-CDSS have been designed and conducted. Highlighting what aspects have been considered and what choices made in previous studies can provide guidance for the design of future experiments. Also, shortcomings in existing studies are identified, and places for improvement can be shown. For the review, we group the design factors and form a framework of ML-CDSS experimental study. In particular, we identify and explore the important individual ML-CDSS domain specific factors that require special attention.

The contribution of this study is that the studies selected for this review compare the performance of clinicians with and without the aid of a ML-CDSS. As far as we know, the studies selected for the previous review studies have compared mainly the performance of clinicians and ML models alone [8–11]. Furthermore, in this study we focus on the practical implementation of a ML-CDSS performance evaluation study. For example, recent artificial intelligence extensions [12, 13] for the clinical trial protocols and reporting guidelines focus on defining the items that should be reported, such as algorithm version and input/output data, not the questions about the practical implementation of experiments.

**Fig. 1:**
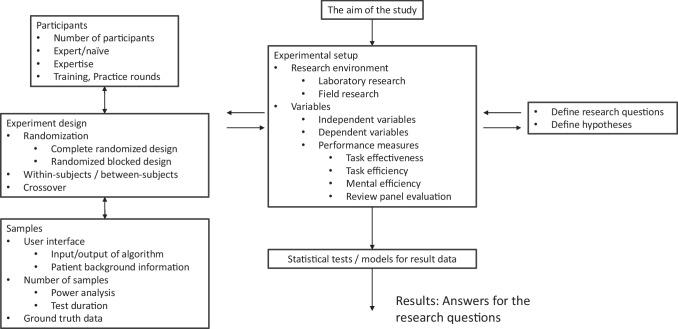
The framework of ML-CDSS performance evaluation study. The factors of the framework are grouped into five groups: experimental setup, participants, experiment design, samples and statistical tests and models for result data

This review study is divided into two parts. Section 2 presents the methods used to search the published ML-CDSS performance evaluation studies and summarizes the selected studies (Table 1). Section 3 forms a framework of ML-CDSS performance evaluation study and groups the factors (Fig. 1), and discusses the important individual design factors.

## Methods

### Search strategies

Our literature search was conducted in PubMed using the combination of search terms (see Appendix). The search was limited to articles published between January 2016 and December 2022.

We included articles if study compared the performance between clinicians with the aid and without the aid of a CDSS, CDSS was based on ML techniques (ML-CDSS), and study was experimental and systematically designed. Articles were excluded if the comparison was between the performance of ML algorithm alone and the performance of a clinician without the CDSS (e.g. [14–19]), or study was observational (e.g. [20, 21]).

On the basis of the above inclusion and exclusion criteria, one author (MN) screened article titles and abstracts and identified eligible articles. The full texts of eligible articles were retrieved. Indistinct samples of this process were resolved by discussion with other authors.

### Results

Following the search process, 1276 citations were retrieved from the database and 1152 articles were excluded based on their titles and abstracts, resulting in 124 articles to be reviewed in detail. In addition, 94 articles were further excluded based on their full text. Finally, 29 studies were included for review.

The data we obtained from each study were year of publication, disease, ML technology, research type (laboratory/field), independent and dependent variables, performance measures, participants (number of subjects, expertise, training), experiment design (randomization, crossover), samples (user interface, number of samples), test duration, ground truth data and performance values.

Table 1 presents top-level figures, such as year of publication, disease, ML technology and expertise of participants. All the selected studies were published between 2018-2022. In many studies, the ML-CDSS was developed to help diagnose cancer (48.28% of the studies). ML technology was based in almost all studies on convolutional neural networks (75.86% of studies). The participants of the studies were most often radiologists (55.17% of the studies).

**Table 1:** Publication year, disease, machine learning technology and expertise of the participants from the studies selected for the review. Diseases: number of cancer ML-CDSS studies: 14 (48.28%); number of fracture ML-CDSS studies: 4 (13.79%); number of other studies: 9 (31.03%); ML technologies: number of CNN techniques: 22 (75.86%); number of RL techniques: 1 (3.45%); number of other techniques: 7 (24.14%); Expertise: number of Anaesthesiologist: 2 (6.90%); number of pathologists: 2 (6.90%); number of endoscopist 4 (13.79%); number of radiologists: 16 (55.17%); number of others: 7 (24.14%). ML = Machine Learning; CNN = Convolution Neural Network; RL = Reinforcement Learning; GMM = Gaussian Mixture Model

Author	Year of publication	Disease	ML technology	Expertise of participants
Dhombres et al. [22]	2019	Pregnancy location	Knowledge based ontology	Obstetrics, gynecology
Lundberg et al. [4]	2018	Hypoxaemia	Gradient boosting	Anaesthesiologist
Steiner et al. [23]	2018	Breast cancer	CNN	Pathologist
Lindsay et al. [1]	2018	Wrist fracture	CNN	Emergency medicine clinician
Kaini et al. [24]	2020	Liver cancer	CNN	Pathologist
Wu et al. [3]	2019	Gastric cancer	CNN, RL	Endoscopist
Wang et al. [25]	2019	Colorectal cancer	CNN	Endoscopist
Bien et al. [26]	2018	Knee injury	CNN + Logistic regression	Radiologist, Orthopedic surgeon
Wijnberge et al. [5]	2020	Hypotension	Logistic regression	Anaesthesiologist
Su et al. [27]	2019	Colorectal cancer	CNN	Endoscopist
Zhou et al. [2]	2020	Rib fracture	CNN	Radiologist
Tajmir et al. [28]	2019	Bone age assessment	CNN	Radiologist
Sim et al. [29]	2019	Lung cancer	CNN, commercial tool	Radiologist
Lee et al. [30]	2020	Thyroid cancer	CNN	Radiologist
Kozuka et al. [31]	2020	Lung cancer	CNN, commercial tool	Radiologist
Jang et al. [32]	2020	Lung cancer	CNN, commercial tool	Radiologist
Cha et al [33]	2019	Muscle-invasive bladder cancer	CNN	Radiologist
Cai et al. [34]	2019	Esophageal cancer	CNN	Endoscopist
Sato et al. [35]	2021	Hip fractures	CNN	Clinician
Yu et al. [36]	2019	Breast cancer	GMM, Random forest	Radiologist
Choi et al. [37]	2021	Thoracic disease	CNN	Radiologist
Choi et al. [38]	2022	Skull fracture	CNN	Radiologist
Shang et al. [39]	2022	SARS-COV-2	CNN	Radiologist
Roller et al. [40]	2022	Graft failure	Gradient boosting	Physicians (internal medicine or nephrology)
Wang et al. [41]	2022	Pancreatic cancer	CNN	Radiologist
Yacoub et al. [42]	2022	Cardiac, pulmonary and musculoskeletal diseases	CNN, commercial tool	Radiologist
Wei et al. [43]	2022	Breast cancer	Commercial tool	Radiologist
Wataya et al. [44]	2022	Lung cancer	CNN	Radiologist
Toda et al. [45]	2022	Lung cancer	CNN, commercial tool	Radiologist, pulmonologist

## Framework for clinical DSS performance evaluation study

Our analysis follows the framework depicted in Fig. 1. The framework, derived from research literature [46, 47], groups the factors of ML-CDSS performance evaluations study in five groups: experimental setup (Section 3.1), participants (Section 3.2), experiment design (Section 3.4), samples (Section 3.3) and statistical tests and models for result data. Next, Sections 3.1–3.4 discusses individual factors of each group.

### Experimental setup

The objective of study defines frames for experimental setup. That is, what is measured (variables that are modified) and where experiment is conducted (research environment). When research environment and independent and dependent variables are fixed, research questions and hypothesis can be refined.

#### Research environment

Research environment can be classified as a field study or a controlled laboratory study or something between them. A field study refers to a natural clinical environment where clinicians use the ML-CDSS. With field studies, not all variables can be controlled for and their effects should be taken into account in the study design. A controlled laboratory study is carried out in an isolated space and time. In laboratory studies, important research variables are modified while other variables are constant.

In the studies in this review, the distinction between a laboratory and a field study was made based on the data type. If a study used retrospective data and participants used ML-CDSS for simulating patient examination, it was considered laboratory research. If a study collected prospective data and ML-CDSS was used as a part of normal patient examination, it was considered field research. Five studies in this review (17.24% of the studies) were classified as field studies (see Table S1 in Supplementary material). In studies [3, 5, 25, 27, 42], clinicians examined consecutive patients randomly with the aid of ML-CDSS or without the aid. The other 25 studies were laboratory studies. These studies used retrospective data that was presented to participants who executed tasks with the aid of ML-CDSS or without the aid. The tasks of these studies simulated patient examinations.

#### Independent and dependent variables

An experimental performance study measures the change of the dependent variable when the level of independent variable is changed. In the studies in this review, the primary independent variable was ML-CDSS aid, which has the two levels: with and without the aid. Dependent variable of the studies was, for example, the number of detected findings, diagnoses estimated by participants or reaction/review time. The primary hypothesis was that the aid of ML-CDSS increases the performance in executing tasks. That is, with the aid of the ML-CDSS, clinicians estimate diagnoses more accurately or detect symptoms faster than without the aid of the ML-CDSS.

Some studies in this review (51.72% of the studies) had more than one independent variable (see Table S1 in Supplementary material). For example, study [1] grouped the participants in the groups of medical doctors and physician assistant. Study [24] grouped the participants in the groups of GI (gastrointestinal) subspecialty, non-GI subspecialty, trainee and pathologist not-otherwise classified. Study [29] grouped the participants in the groups of resident or chest radiologists. Study [30] groups the participants in the groups of trainee or staff radiologist. Study [24] measured the effect of tumor grade for the clinicians’ performance. Study [33] measured the effect of the difficulty of chemotherapy response evaluation for the clinicians’ performance. These additional independent variables answered to the research question if clinician’s professional level or the difficulty of the task had an effect on the clinician’s performance to execute tasks with or without the aid.

#### Performance measures

If participants perform tasks more effectively or efficiently at one level of the independent variable than at the other, the value of the dependent variable changes. The change is quantized by performance measures. Study [48] grouped the performance measures in three categories: task effectiveness, task efficiency and mental efficiency. In addition to this, we defined a fourth category: review panel evaluation. Review panel evaluation measures are values that are scored after the study by an external expert panel.

Table 2 groups the dependent variables and performance measures of the studies in this review into four performance measure categories. 24 studies (82.76% of the studies) in this review, used task effectiveness measures (see Table S1 in Supplementary material). Task effectiveness measures indicate the ratio of correctly classified or evaluated samples to total number of samples. These measures identify whether the ML-CDSS assisted participants to perform tasks more effectively. For example, the accuracy of diagnoses (e.g., early pregnancy, cancer diagnose, knee injury, wrist or rib fractures, bone age) was an often-used performance measure [22, 24, 26, 28–30]. Also, the performance measures of sensitivity and specificity [1, 2, 23, 26, 31, 41, 43, 44] and AUC value (area under curve) [4, 32, 33, 37, 38, 40, 43–45] were often used.

11 studies (37.93% of the studies) in this review, used task efficiency measures (see Table S1 in Supplemenary material). Task efficiency measures indicate if ML-CDSS aided clinician to execute tasks more efficiently. These measures are related to examination time or the number of repetitions, detections or incidents. For example, studies [2, 3, 22, 23, 25, 27, 32, 41, 42, 44] measured time to execute tasks (e.g., scan duration, review time, reaction time, reading time). Studies [3, 5, 22, 25] measured the numbers of detections (e.g., number of polyps, number of unobserved sites), repetitions (e.g., number of scan images) and incidents (e.g., number of treatments or hypotensive events).

Detection rate is a typical value derived from the numbers of detections. For example, study [25] calculated the detection rates of polyps/adenomas per sample. That is, how many polyps/adenomas clinicians found from one colonoscopy patient with the aid of ML-CDSS or without the aid.

Mental efficiency measures indicate required mental resource or confidence to perform a task. Mental efficiency can be measured with a self-reported questionnaire, which collects numerical values or comments. Three studies in this review [23, 30, 32] measured mental efficiency values (see Table S1 in Supplementary material). Study [23] used the scale of 0-100 to rate obviousness, when the task was to decide the category of negative, isolated tumor cells, micrometastasis, or macrometastasis from digitized slides from lymph node sections. Study [30] used the scale of 1-5 when the task was to measure the level of confidence in identifying cervical lymph node metastasis. Study [32] used the scale of 1-100 when the task was to measure the level of confidence in detecting potential malignant lung lesions.

Review panel evaluation relates to an external expert panel which performs a post-hoc evaluation for the results/documentation of a task. Two studies in this review used review panel evaluation measures (see Table S1 in Supplementary material). For example, in study [22] an external expert panel evaluated the trustworthiness of documentation produced by the participants of an experiment. The task of the participants was to diagnose early pregnancy and pregnancy location from ultrasound imaging. In study [3], an external expert panel evaluated the completeness of photo documentation produced by the participants who conducted esophagogastroduodenoscopy examinations.

**Table 2:** Performance measures and dependent variables from the studies of this review for evaluating clinicians with or without the aid of ML-CDSS were categorized into the groups of task effectiveness, task efficiency, mental efficiency and review panel evaluation. AUC = Area Under Curve, RMSE = Root Mean Square Error

	Performance measure	Dependent variable	Authors
Task effectiveness	Accuracy, sensitivity, specificity	Pregnancy location and diagnosis	[22]
		Cancer diagnosis/detection	[23, 24, 29–32, 34, 36, 41, 43, 44]
		Knee injury diagnosis	[26]
		Fracture diagnosis	[1, 2, 35, 38]
		Bone age (one year range)	[28]
		SARS-CoV-2 diagnosis	[39]
		Abnormal finding	[45]
	AUC	A relative risk of hypoxaemia	[4]
		Confidence rate if malignant lung lesion is detected	[32, 45]
		Likelihood of complete response of chemotherapy	[33]
		Localizing thoracic abnormalities	[37]
		Likelihood of fracture	[38]
		Likelihood of graft failure	[40]
		Likelihood of presence of the 15 characteristics (pulmonary nodules nodules)	[44]
		Dichotomized pattern of benign or malignant breast masses	[43]
	RMSE	Bone age	[28]
Task efficiency	Time (avg)	Reading, review, withdrawal, scan, inspection, diagnosis time	[1–3, 5, 22, 23, 25, 27, 32, 41, 42, 44]
		Time-weighted average of hypotension, total time with hypotension, percentage of time spent with hypotension during surgery	[5]
	Number of repetitions	Number of scans	[22]
	Number of detections	Number of detected polyps/adenomas	[25, 27]
		Number of unobserved sites in patient	[3]
		Number of chest CT recommendations	[32]
	Number of incidents	Number of hypotensive events per patient	[5]
		Number of treatments per patient	[5]
Mental efficiency	Obviousness score	Obviousness rate of breast cancer lymph node	[23]
	Confidence score	Level of confidence in identifying cervical LNM	[30]
		Confidence rate for detected malignant lung lesion	[32]
Review panel evaluation	Trust	Trust score (results of simulated ultrasound imaging)	[22]
	Quality	Quality of image set (results of simulated ultrasound imaging)	[22]
	Completeness	Completeness of photo documentation (real time esophagogastroduodenoscopy)	[3]

#### Statistical tests

Statistical tests are used to prove the statistical significance of the difference between the values of performance measure for the different levels of the independent variable. The selected performance measure defines the requirements for statistical tests. Different tests are used for continuous, category and count data. In many studies, t-test was used because the output of performance measures was continuous value [4, 22, 26, 27, 39–42]. Also, non-parametric Mann Whitneu U test was used in some studies [3, 44]. For the categorical outputs, for example, the exact McNemar or chi-squared tests were used [3, 5, 22, 26, 27, 41–43].

### Participants

With traditional randomized clinical trials, the patients undergoing medical examination are the participants of the study. With ML-CDSS performance experiment studies, clinicians who execute experimental tasks are the participants of the study.

In general, experimental study participants are classified as naïve or experts. Naïve participants do not have deep understanding or experience in the domain whereas expert participants do. The participants in the all studies in this review were experts (Table 1). They were pathologists [23, 24], endoscopists [3, 25, 27], radiologists [26, 28–33, 36, 37, 41–44], orthopedic surgeons [26], internal medicine and nephrology physicians [40], emergency physicians [38] or anaesthesilogists [4, 5].

The important study design question is the number of participants. In the studies reviewed, the number of participants was mainly between 2-16 (see Table S2 in Supplementary material). Studies [1] and [35] were exceptions with 40 and 31 participants. According to recommendations, the number of naïve participants should be more than 15 [49] or 20 [50]. Statistically significant results are possible to achieve with lower numbers of expert participants than with naïve participants. According to the study [51], the number of expert participants should be 10-15. However, none of the studies in this review discussed how they chose the number of expert participants.

### Samples

#### User interface

All data (patient information) of the experiment is presented via the user interface (UI) for participants and the values of dependent variables are entered using the input elements of the UI. We identified two patient information types presented in the UI: patient background information and decision support information. Patient background information means patient-related medical knowledge, such as medical history and results from physical examinations, laboratory or imaging findings. Decision support information means the output information of the ML algorithm incorporated in the ML-CDSS.

Table 3 presents patient background and decision support information that was presented for participants in the studies in this review. We found that 22 studies in this review presented only decision support information on UI, such as heat maps or bounding boxes on medical image [1, 2, 22–24, 26, 29–39, 42–45], but no patient background information on the UI. Only seven studies [3–5, 25, 27, 40, 41] presented patient background information on the UI.

We further divided decision support information into two types:

- Category support information: a predicted probability/category of diagnosis/state of patient
- Guidance support information: an instructional guidance for participants to execute patient examination comprehensively or better.

In 25 studies in this review (86.2% of the studies), decision support information belonged to the category group (Table 3). In many of these cases, decision support information was presented as heat maps on medical images. For example, in study[1] if the ML algorithm found a fracture, heat map was used for showing the location of the fracture and the confidence of the model’s prediction. Study [23] visualized confidence that tissue contains tumor by using cyan and green rectangles on images. Study [26] highlighted regions on an image that were important for the model’s knee injury classification decision.

In four studies [3, 22, 25, 27], decision support information belonged to the guidance group (Table 3). The aid tested in these studies was instructional guidance for participants to execute a task more efficiency or comprehensive. For example, in study [3] a virtual stomach model was presented to guide endoscopist to find blind spots. In studies [25, 27] bounding boxes were presented on the video image for showing the locations of polyps. Furthermore, audio prompts were played to help tune withdrawal speed or to alarm for potential polyps. In study [22] the ML-CDSS presented keyword suggestions for participants for selecting reference images from a database.

**Table 3:** The patient background information and decision support information presented on the ML-CDSS user interface. Patient background information means patient-related medical knowledge, such as medical history and results from physical examinations, laboratory or imaging findings. Decision support information means the output information of the machine learning algorithm incorporated in the ML-CDSS. Decision support information is grouped into two types: Category support information and Guidance support information. Category support information presents a predicted probability/category of diagnosis/state of patient. Guidance support information presents an instructional guidance for participants to execute patient examination comprehensively or better

Author	Task	Patient background information	Decision support information	Decision support: Category/Guidance
Dhombres et al. [22]	Ultrasound examination for early pregnancy diagnostic	N/A	Guided keywords selection, presentation of reference images for the selected keywords and suggestions of additional views	Guidance
Lundberg et al. [4]	Hypoxaemia risk prediction	Patient record data	Risk of hypoxaemia in the next five minutes and explanations	Category
Steiner et al. [23]	Lymph node image classification	N/A	Regions on the lymph node image were highlighted based on the algorithm predictions.	Category
Lindsey et al. [1]	Wrist fracture detection	N/A	Regions on the radiograph image were highlighted based on the algorithm predictions	Category
Kiani et al. [24]	Subtype of primary liver cancer classification	N/A	Regions on the image crop of H &E stained digital WSI were highlighted based on the algorithm predictions	Category
Wu et al. [3]	EGD study and photodocumentation	All patient information was available (field study)	Virtual stomach model for monitoring blind spots (sites not detected yet by the endoscopist)	Guidance
Wang et al. [25]	Colonoscopy examination for polyp and adenoma detection	All patient information was available (field study)	The detected polyps are presented with a hollow blue tracing box with a sound alarm	Guidance
Bien et al. [26]	Knee injury classification	N/A	Regions on the MRI were highlighted based on the algorithm predictions.	Category
Wijnberge et al. [5]	Elective noncardiac surgical procedure with the early warning system of intraoperative hypotension	All patient information was available (field study)	If the predicted risk of hypotension was high, sound and flickering light were presented. Mixture of variable information provided information about the underlying cause of the predicted hypotension.	Category
Su et al. [27]	Colonoscopy examination for polyp and adenoma detection	All patient information was available (field study)	(1) a real-time timer; (2) an audio prompt to remind to slow down the withdrawal speed and reexamine certain colonic segments when unstable or blurry frames were detected; (3) an audio prompt to persuade to clean the mucosa or suction liquid pools if needed; (4) a bounding box showing the location of a polyp	Guidance
Zhou et al. [2]	Rib fracture detection and classification	N/A	CT image with rectangular boxes indicating predicted fractures	Category
Tajmir et al. [28]	Bone age assessment	N/A	Bone age prediction and attention map on the radiograph	Category
Sim et al. [29]	Malignant pulmonary nodule detection from chest radiographs	N/A	Dotted circles were presented on the radiograph based on the algorithm prediction	Category
Lee et al. [30]	Cervical lymph node metastasis diagnosis from CT images	N/A	Regions on the CT image were highlighted based on the algorithm predictions	Category
Kozuka et al. [31]	Pulmonary nodule detection from CT images	N/A	Marks, density, major axis, and the volume of detected nodules on the CT image	Category
Jang et al. [32]	Pulmonary nodule detection from radiograph	N/A	Color coded map indicating a probability that a radiograph contains a malignant nodule	Category
Cha et al [33]	Chemotherapy response detection	N/A	Pre- and post-treatment CT scans were presented side-by-side with likelihood score of response	Category
Cai et al. [34]	Esophageal squamous cell carcinoma (ESCC) detection	N/A	The lesions on the image were marked with a square based on the algorithm predictions	Category
Sato et al. [35]	Hip fracture detection	N/A	Regions on the radiograph image were highlighted based on the algorithm predictions	Category
Yu et al. [36]	Breast lesions diagnostic	N/A	Regions on the radiograph image were highlighted based on the algorithm predictions	Category
Choi et al. [37]	Thoracic abnormalities detection from chest radiographs	N/A	Regions on the radiograph image were highlighted based on the algorithm predictions	Category
Choi et al. [38]	Skull fracture detection	N/A	Regions on the radiograph image were highlighted based on the algorithm predictions (+ probablity prediction)	Category
Shang et al. [39]	SARS-CoV-2 virus diagnosis from lung ultrasonography	N/A	Regions on the LUS image were highlighted based on the algorithm predictions (+ classification result)	Category
Roller et al. [40]	Detection of graft failure	Complete patient history, including text notes, medical reports, laboratory tests	Risk scores, relevant features which influence the decision of the risk score	Category
Wang et al. [41]	Pancreatic lesion diagnostic	Radiological characteristics	Risk prediction	Category
Yacoub et al. [42]	Chest CT interpratation	N/A	Analysis of finding, including labeling, segmenting and measuring normal structures as well as detecting, labeling and measuring abnormalities	Category
Wei et al. [43]	Breast lesions diagnostic	N/A	Risk prediction	Category
Wataya et al. [44]	Pulmonary nodule diagnostic from radiograph	N/A	Presents characterization results from lung nodule and three candidate radiology reports	Category
Toda et al. [45]	Detection of pulmonary nodules, masses and consolidation	N/A	Detects pulmonary nodules/masses and consolidation and marks the areas of the lesions	Category

#### Number of samples

In the studies in this review the term sample refers to an entity being examined by the participant, i.e. a patient or patient data, such as imaging data, vital signs or other patient record data. Many factors affect the required number of samples, such as evaluation time of one sample, availability of participants and the sensitivity of dependent variables for the changes of the independent variable.

Power calculation is a traditional method for defining the minimum number of samples required for statistically significant results. Seven studies [3, 5, 25, 27, 37, 40, 42] of this review used and reported power calculations (see Table S3 in Supplementary material). The numbers of samples required according to the power calculation were between 30 and 651.

If the number of samples is high and test duration becomes too long, participants tire, and the quality of collected data suffers. Also, new participants can be difficult to recruit for long experiments. Only one study [23] of this review discussed or documented test duration: the test duration was 3 hours including training, instructions, and breaks (see Table S3 in Supplementary material).

Also, training and practice rounds lengthen the duration of experiment. Although, the training and practice rounds are important for reliable results, only five studies in this review reported that they conducted some training before the experiment (see Table S3 in Supplementary material). Study [23] presented five and study [24] four training samples for the participants. Study [22] presented a 2 minutes video and conducted a 10 minutes hands on session before the experiment. In study [44], the participants received training on the definition of characteristics of the platform before the experiment. In study [42], the participants received training on interpretating the platform and used the platform for at least 30 days.

#### Ground truth data

One important study design question is how ground truth (GT) data is produced. GT data must be a close estimate for the true values of samples. In this study we divided GT data production methods into four types: (1) Majority vote, (2) High expertise, (3) Many data sources, and (4) Numerical data (see Table S3 in Supplementary material).

Majority vote method assumes that the majority opinion of a review group is GT for a sample. In many settings, majority vote and high expertise methods are used together. That is, the group of highly experienced clinicians is used for voting. For example, in study [23] GT data was produced by the majority vote of three experts (US board certified pathologists, > 7 years of experience). In that study and also in the studies [1–3, 26, 29, 31, 34, 45, 52], GT method was both majority vote and high expertise.

If the number of experts used for generating GT data is smaller than three, the method is classified as high expertise only, not majority vote. For example, in study [22] the documents produced by participants were reviewed and scored by two senior experienced ultrasound operators.

The method of deriving GT data from many data sources combines information from different sources that are not available for participants. These data sources are, e.g., patient records and other longitudinal patient tracking data. The method is viable in particular for retrospective studies where longitudinal patient data after patient examination is available. For example, in study [4] the hypoaxemia states of the patients retrieved from patient records were considered GT data. Other examples of the GT method of many data sources are studies [24, 26, 28, 30, 33, 34, 36, 37, 39, 40, 43]. In study [26], expert group produced GT data by using all DICOM series, clinical history and follow-up exams of samples. In study [28], experts had access to machine learning attention maps, machine learning bone age scores, and the clinical reports to define the bone age from radiographs.

Whereas usually GT data represents the "true diagnosis", GT method of numerical data refers to settings where the evaluation is not about the correctness of the finding, but rather number of findings or the speed of decision making. For example, in studies [25, 27] the performance was calculated based on the number of detected and analyzed polyps/adenomas. Higher number of detected polyps/adenomas was evaluated to be a better result. In study [5] shorter hypotension time of the patient and a shorter reaction time of the participants were better results. In study [42] shorter interpretation time of the chest computer tomography image was better result.

Post-hoc GT data means that ground truth values are produced after the experiment. That is, no prior true values for samples exist. For example, in study [3], after experiment, two seniors (1–5 years of experience) and three experts (>5 years of experience) reviewed and scored endoscopy videos and documentation generated by the participants. It should be noted, that the concepts of post-hoc GT and review panel evaluation measure group (Table 2) are similar or same.

### Experiment design

#### Randomization and within-subjects/between-subject design

Traditional experiment design can be, e.g., complete randomized design (CRD) or randomized block design (RCBD). It should be noted that CRD and RCBD concepts differ somewhat between traditional clinical trials and ML-CDSS experiment studies. In the first case, patients are the participants of the study who are randomized into groups (yes/no treatment) forming the independent variable. In the latter case, clinicians are the participants of the study, and the independent variable to be randomized is with/without the aid of ML-CDSS.

The CRD divides clinicians randomly into two groups. One group executes tests with the aid of a ML-CDSS and the other group without it. In RCBD, first the heterogenous participant group is divided into homogeneous same-size sub-groups (blocks). Next, examinations with or without the aid are randomized for different blocks. In that way, the same number of participants from different blocks execute the experiment with and without the aid of a ML-CDSS. This can eliminate variance sources from confounding factors, such as professional level of participants. That is, RCBD ensures that with/without aid condition has, for example, an equal proportion of different professional levels. As a result, differences between the conditions cannot be attributed to professional level.

Furthermore, the experiment design can be classified as within-subject or between-subject design. In the between-subject design, one clinician conducts the experiment always at the same level of the independent variable. In the within-subject design, all levels of independent variable are presented for all clinicians. That is, between-subject design divides clinicians into two groups: one group executes tests with the aid and other group without the aid. Within-subject design presents the with aid and without aid settings for all clinicians. That is, all clinicians evaluate the same sample twice (with and without aid) or part of samples with the aid and part of samples without the aid. In this review, 82.8% of the studies applied within-subject experiment design (see Table S4 in Supplementary material). For example, studies [1, 22, 29, 31, 45] presented all samples without the aid first and then with the aid for all participants. Study [23] presented samples randomly with or without the aid first for all participants and then the opposite for the second round. Studies [2, 5, 28, 30, 43] presented one sample first without the aid of ML-CDSS and then with the aid.

#### Crossover

Crossover is an important concept for evaluating the performance of the aid of ML-CDSS. Crossover design executes two or more experiment sessions for each participant. For example, in the first experiment session, participants evaluate half of the samples with the aid and half of samples without the aid. Then, after a washout period, in the second session, participants evaluate the same samples but now with aid, if originally evaluated without aid, and vice versa. It is assumed that the washout period decreases the memory footprint from the first session. It should be noted, that crossover design is limited for laboratory studies in which retrospective data is used.

Twelve studies [22–24, 26, 29, 31, 32, 34, 38, 44] in this review (41.4% of the studies) used a crossover design (see Table S4 in Supplementary material). The length of the washout period varied. In studes [2, 22] the washout period was two months. In study [29] the washout period was only 2-6 hours. In other studies, the washout period was from 7 to 28 days. When planning the length of the washout period, it is important to note that participants may recall samples for a long period after the experiment, particularly the difficult samples. Study [53] recommended that the washout period should be at least 2 weeks. On the other hand, with a long washout period, the participant’s diagnostic criteria could have changed over time. For example, participants could have gained more experience or changed their attitude toward diagnostic criteria [54].

## Discussion

The aim of this review study was to summarize experimental study design factors for measuring the performance of clinicians with or without the aid of a ML-CDSS. The key factors were performance measures, user interface, ground truth data, samples and participants.

**Fig. 2:**
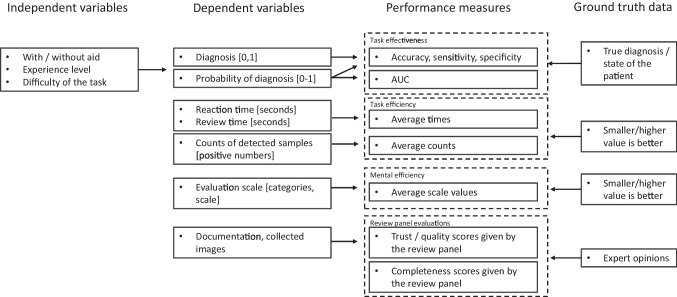
Experimental performance study of clinical decision support system aims to prove that by changing the independent variable the dependent variables change. Performance measures are used to measure the significancy of the change. Performance measures are calculated by comparing the values of dependent variables and ground truth. In this study we grouped the performance measures into the groups of task effectiveness, task efficiency, mental efficiency and review panel evaluation. Different ground truth data types are used for different performance measures

Figure 2 shows dependencies between the selection of proper performance measure and dependent variables and ground truth. First, dependent variables (input requested from the participant) determine which performance measures can be calculated. With the task effectiveness measures (such as accuracy, sensitivity, specificity and AUC), input value is binary [0,1] or a probability/continuous value. If the AUC value is the preferred performance measure, then the input should be a probability/continuous value. Accuracy, sensitivity and specificity performance measures can be calculated from binary inputs. It’s important to note, that probability/continuous values can be harder for participants to estimate than binary states. Second, the research environment (retrospective vs. prospective study or laboratory vs. field research) also affects selectable performance measures. With prospective research settings, no task effectiveness measures (such as AUC, accuracy, sensitivity, specificity) can be used, because no exact numerical GT values are available. Task effectiveness measures require exact GT values, such as true diagnosis or patient state. That is, for the prospective research settings, the performance measures of task efficiency, mental efficiency or review panel evaluations should be used.

Different performance measures provide different information about the ML-CDSS. Task effectiveness and task efficiency measures calculate simple numerical or ratio values (e.g. accuracy level or detection ratio). If more deep or subjective evaluations, such as benefits or pitfalls of the system, are required, the use of mental efficiency measures should be considered. For example, mental efficiency measures can be used for evaluating the difficulty of the task or participant’s confidence or what are situations when ML-CDSS interferes decision making. Answers can be given on a numerical scale or in open answers. For example, study [55] used a method of "thinking-aloud-test" in which the participants communicate their thoughts aloud while interacting with the system. For the situations when the outputs of participants are difficult to quantify, such as written reports or other documentation, the evaluation of review panel measures can be the proper choice. However, review panel evaluation measures are expensive to use, when the number of participants is high.

A key step of designing the elements of UI is to determine available patient information. In this review, we divided patient information into the groups of patient background and decision support. Patient background information is all relevant medical knowledge about the patients. Decision support information is based on the output of the incorporated ML algorithm. First step of designing the UI is to review information available at a normal patient examination. That is, patient background information that clinicians use for planning treatments and interventions. We found that many studies in this review presented only decision support information on UI, but no patient background information on the UI. It should be noted that the results of laboratory type studies are comparable to normal patient examinations only when all patient’s background information relevant to the task is presented in the UI.

The design of the number of participants and samples and the design of duration of test are interdependent. The required number of samples depends on how large the difference is between the levels of dependent variable. If the difference is small, the number of samples should be higher for significant results. Power analysis can be used for approximating number of samples. The total number of samples can be increased either by increasing the number of participants or by increasing the number of samples evaluated by one participant. It should be remembered, though, that when the number of samples evaluated by one participant is increased, experiment duration lengthens. Too long an experiment causes fatigue, which lowers the quality of input values. It is also important to document all relevant information about the samples and participants. Only one study [23] in this review documented the duration of the experiment. One rule of thumb for determining the number of samples is to limit the duration of the test to 30 minutes. Then by calculating an average time to evaluate one sample, a maximum number of samples can be calculated. The number of participants should be high enough to produce significant differences between the samples.

In addition to the factors of experimental design, there are other important issues involved in the practical implementation of ML-CDSS experiments that should be recognized. For example, the accuracy of the ML method incorporated in ML-CDSS affects the performance of the clinicians. For example, in study [24], when the ML algorithm predicted correct diagnoses, ML-CDSS improved the accuracy of the clinicians. When the predictions were incorrect, ML-CDSS significantly decreased their accuracy.

The conclusions of the experiment should analyze whether the use of a ML-CDSS caused false negatives. That is, positive samples were missed because of the suggestions of the ML-CDSS. A reason can be that clinicians relied more on the ML-CDSS than their own conclusions. False negatives can be related to the low accuracy of ML algorithm, but may also be related to the information presented on UI. For example, if explanations for the factors affecting the predictions of the ML algorithm are presented and are interpretable in the UI, the clinician can interpret whether the prediction is reliable and whether it should be taken into account in the decision making. One important research question of ML-CDSS performance evaluations presented in recent studies is how providing explanations of ML model results and factors affecting it benefits decision making [48, 56, 57].

Finally, when drawing conclusions from the results of the experiment, it is important to keep in mind that experimental environment often does not correspond to a real clinical environment. For example, in a laboratory study of ML-CDSS, there are no unrelated distractions, nor are there other examinations requiring the attention of clinicians. This may increase the performance of clinicians to decide the condition of patient.

### Limitations

The coverage of the ML-CDSS studies selected for this review may be incomplete. For example, we did not review conference abstracts or studies written in a language other than English. In addition, publication biases may occur because studies that report results showing that a ML-CDSS increased the performance are more likely to be published more frequently.

## Conclusions

The aim of this review study was to analyse how experimental studies for measuring the performance of clinicians with or without the aid of ML-CDSS have been conducted. We explored key design factors and reviewed the choices made in previous studies regarding the factors. For example, dependent variables of experiment setup and available patient data determine performance measures that can be calculated. If the performance is measured by AUC values, probability/continuous input values (dependent variables) and retrospective patient data are required. In some studies more deep or subjective information, than just numerical values, were collected by mental efficiency type measures. The number of samples, number of participants and the duration of experiment are interrelated. The number of samples should be high enough to produce statistically significant results. However, increasing the number of samples per participant and thereby increasing the duration of experiment, can cause fatigue, which can lower the quality of input values. In many studies no patient background information was available for the participants. Such experiment setups differ from a real world patient examination and can bias the results.

### Supplementary Information

Below is the link to the electronic supplementary material.

Supplementary file1 (PDF 124 kb)

## Data Availability

Not applicable.

## Appendix: Search syntax for the Pubmed

("Machine Learning*"[Mesh] OR "Deep Learning"[MeSH] OR "Neural Networks, Computer*"[Mesh] OR scan assistant[Title/Abstract] OR “deep learning” [Title/Abstract] OR "random forest"[Title/Abstract] OR "support vector machine" [Title/Abstract] OR "decision tree"[Title/Abstract] OR "gradient boosting"[Title/Abstract])

AND

("Proof of Concept Study"[Mesh] OR "ROC Curve"[Mesh] OR “Prospective Studies” [Mesh] OR “with and without” [Title/Abstract] OR examination[Title/Abstract]) OR "controlled experiment"[Title/Abstract] OR “unblinded randomized clinical trial” [Title/Abstract] OR “prospective randomized controlled study” [Title/Abstract])

AND

("Decision Support Systems, Clinical"[Mesh] OR "Image Interpretation, Computer-Assisted"[Mesh] OR "Image Interpretation, Computer-Assisted/statistics and numerical data"[MAJR] OR "Image Processing, Computer-Assisted/methods"[MAJR] OR "clinical understanding" [Title/Abstract] OR “computer-aided diagnosis” [Title/Abstract] OR “early warning system” [Title/Abstract])

NOT

(Comment[Publication Type] OR editorial[Publication Type] OR letter[Publication Type] OR case reports[Publication Type])
