# Supplementary material for: Systematic review of the performance evaluation of clinicians with or without the aid of machine learning clinical decision support system
Source: Health Technol (Berl). 2023 Jun 13:1–14. Online ahead of print. doi: 10.1007/s12553-023-00763-1 (PMC10262137; doi:10.1007/s12553-023-00763-1)
Supplement: Supplementary file 1 — Supplementary file1 (PDF 124 kb) [file 12553_2023_763_MOESM1_ESM.pdf]

Supplementary material: Systematic review of the performance evaluation of clinicians with or without the aid of machine learning clinical decision support system

Table S1: Statistics of experimental setup (research environment, independent variables and performance measures) for the studies selected for the review

	Research environment		Independent variable			Performance measures			
Author	Laboratory	Field	The aid of ML-CDSS	Experience level	Case difficulty	Task effectiveness	Task efficiency	Mental efficiency	Review panel evaluation
Dhombres et al. [S1]	x		x			x	x		x
Lundberg et al. [S2]	x		x			x			
Steiner et al. [S3]	x		x			x	x	x	
Lindsay et al. [S4]	x		x	x		x	x		
Kaini et al. [S5]	x		x	x	x	x			
Wu et al. [S6]		x	x				x		x
Wang et al. [S7]		x	x				x		
Raipurker et al. [S8]	x		x			x			
Wijnberge et al. [S9]		x	x				x		
Su et al. [S10]		x	x				x		
Zhou et al. [S11]	x		x			x	x		
Tajmir et al. [S12]	x		x			x			
Sim et al. [S13]	x		x	x		x			
Lee et al, [S14]	x		x	x		x		x	
Kozuka et al. [S15]	x		x			x			
Jang et al. [S16]	x		x	x		x		x	
Cha et al [S17]	x		x		x	x			
Cai et al. [S18]	x		x	x		x			
Sato et al. [S19]	x		x	x		x			
et al. [S20]	x		x			x			
Choi et al. [S21]	x		x	x		x			
Choi et al. [S22]	x		x	x		x			
Shang et al. [S23]	x		x	x		x			
Roller et al. [S24]	x		x	x		x			
Wang et al. [S25]	x		x	x		x	x		
Yacoub et al. [S26]		x	x				x		
Wei et al. [S27]	x		x	x		x			
Wataya et al. [S28]	x		x	x		x	x		
Toda et al. [S29]	x		x	x		x			
Sum	24	5	29	15	2	24	11	3	2
Percentage	82.76%	17.24%	100.00%	51.72%	6.90%	82.76%	37.93%	10.34%	6.90%

Table S2: Statistics of participants for the studies selected for the review

Author	Number of participants	Experts	Naive
Dhombres et al. [S1]	2	x	
Lundberg et al. [S2]	5	x	
Steiner et al. [S3]	6	x	
Lindsay et al. [S4]	40	x	
Kaini et al. [S5]	11	x	
Wu et al. [S6]	6	x	
Wang et al. [S7]	8	x	
Raipurker et al. [S8]	9	x	
Wijnberge et al. [S9]	N/A	x	
Su et al. [S10]	6	x	
Zhou et al. [S11]	5	x	
Tajmir et al. [S12]	3	x	
Sim et al. [S13]	12	x	
Lee et al, [S14]	7	x	
Kozuka et al. [S15]	2	x	
Jang et al. [S16]	9	x	
Cha et al [S17]	13	x	
Cai et al. [S18]	16	x	
Sato et al. [S19]	31	x	
et al. [S20]	2	x	
Choi et al. [S21]	6	x	
Choi et al. [S22]	5	x	
Shang et al. [S23]	6	x	
Roller et al. [S24]	8	x	
Wang et al. [S25]	3	x	
Yacoub et al. [S26]	3	x	
Wei et al. [S27]	4	x	
Wataya et al. [S28]	15	x	
Toda et al. [S29]	12	x	
Sum		29	0
Percentage	9.11	100%	0%

Table S3: Statistics of samples and ground truth data for the studies selected for the review.

	Samples					GT data prodcution			
Author	Total number of samples	Per participants	Power analysis	Test duration documented	Practice round	Majority vote	High expertise	Many data sources	Numerical data
Dhombres et al. [S1]	64				x		x	x	
Lundberg et al. [S2]	198							x	
Steiner et al. [S3]	140			x	x	x	x		
Lindsay et al. [S4]	300					x	x		
Kaini et al. [S5]	160				x		x	x	
Wu et al. [S6]	303		x			x	x		
Wang et al. [S7]	1130	148	x						x
Raipurker et al. [S8]	120					x	x	x	
Wijnberge et al. [S9]	68		x						x
Su et al. [S10]	623		x						x
Zhou et al. [S11]	63					x	x		
Tajmir et al. [S12]	280						x	x	
Sim et al. [S13]	800	67				x	x		
Lee et al, [S14]	242						x	x	
Kozuka et al. [S15]	120					x	x		
Jang et al. [S16]	180						x	x	
Cha et al [S17]	123						x	x	
Cai et al. [S18]	52					x	x	x	
Sato et al. [S19]	300						x		
et al. [S20]	274						x	x	
Choi et al. [S21]	244		x				x	x	
Choi et al. [S22]	95						x		
Shang et al. [S23]	1307							x	
Roller et al. [S24]	120	30	x	x				x	
Wang et al. [S25]	102						x		
Yacoub et al. [S26]	390	130	x		x				x
Wei et al. [S27]	192							x	
Wataya et al. [S28]	101				x		x		
Toda et al. [S29]	200					x	x		
Sum			7	2	5	9	21	14	4
Percentage / average	285.8965517	93.75	24.14%	6.90%	17.24%	31.03%	72.41%	48.28%	13.79%

Table S4: Statistics of participants for the studies selected for the review

	Study design		
Author	Within subject	Cross-over design	Washout period
Dhombres et al. [S1]	x	x	60
Lundberg et al. [S2]			
Steiner et al. [S3]	x	x	28
Lindsay et al. [S4]	x		
Kaini et al. [S5]	x	x	14
Wu et al. [S6]			
Wang et al. [S7]			
Raipurker et al. [S8]	x	x	10
Wijnberge et al. [S9]			
Su et al. [S10]			
Zhou et al. [S11]	x	x	60
Tajmir et al. [S12]	x	x	N/A
Sim et al. [S13]	x	x	0.17
Lee et al, [S14]	x		
Kozuka et al. [S15]	x	x	14
Jang et al. [S16]	x	x	28
Cha et al [S17]	x		
Cai et al. [S18]	x	x	28
Sato et al. [S19]	x		28
et al. [S20]	x		
Choi et al. [S21]	x		
Choi et al. [S22]	x	x	14
Shang et al. [S23]	x		
Roller et al. [S24]	x		
Wang et al. [S25]	x		
Yacoub et al. [S26]	x		
Wei et al. [S27]	x		
Wataya et al. [S28]	x	x	7
Toda et al. [S29]	x		
Sum	24	12	
Percentage / average	82.76%	41.38%	24.3

References

[S1] Dhombres F, Maurice P, Guilbaud L, Franchinard L, Dias B, Charlet J, et al. A novel intelligent scan assistant system for early pregnancy diagnosis by ultrasound: clinical decision support system evaluation study. Journal of medical Internet research. 2019;21(7):e14286.

[S2] Lundberg SM, Nair B, Vavilala MS, Horibe M, Eisses MJ, Adams T, et al. Explainable machine-learning predictions for the prevention of hypoxaemia during surgery. Nature biomedical engineering. 2018;2(10):749–760.

[S3] Steiner DF, MacDonald R, Liu Y, Truszkowski P, Hipp JD, Gammage C, et al. Impact of deep learning assistance on the histopathologic review of lymph nodes for metastatic breast cancer. The American journal of surgical pathology. 2018;42(12):1636.

[S4] Lindsey R, Daluiski A, Chopra S, Lachapelle A, Mozer M, Sicular S, et al. Deep neural network improves fracture detection by clinicians. Proceedings of the National Academy of Sciences. 2018;115(45):11591–11596.

[S5] Kiani A, Uyumazturk B, Rajpurkar P, Wang A, Gao R, Jones E, et al. Impact of a deep learning assistant on the histopathologic classification of liver cancer. NPJ digital medicine. 2020;3(1):1–8.

[S6] Wu L, Zhang J, Zhou W, An P, Shen L, Liu J, et al. Randomised controlled trial of WISENSE, a real-time quality improving system for monitoring blind spots during esophagogastroduodenoscopy. Gut. 2019;68(12):2161–2169.

[S7] Wang P, Berzin TM, Brown JRG, Bharadwaj S, Becq A, Xiao X, et al. Real-time automatic detection system increases colonoscopic polyp and adenoma detection rates: a prospective randomised controlled study. Gut. 2019;68(10):1813–1819.

[S8] Bien N, Rajpurkar P, Ball RL, Irvin J, Park A, Jones E, et al. Deep-learning-assisted diagnosis for knee magnetic resonance imaging: development and retrospective validation of MRNet. PLoS medicine. 2018;15(11):e1002699.

[S9] Wijnberge M, Geerts BF, Hol L, Lemmers N, Mulder MP, Berge P, et al. Effect of a machine learning–derived early warning system for intraoperative hypotension vs standard care on depth and duration of intraoperative hypotension during elective noncardiac surgery: the HYPE randomized clinical trial. Jama. 2020;323(11):1052–1060.

[S10] Su JR, Li Z, Shao XJ, Ji CR, Ji R, Zhou RC, et al. Impact of a real-time automatic quality control system on colorectal polyp and adenoma detection: a prospective randomized controlled study (with videos). Gastrointestinal endoscopy. 2020;91(2):415–424.

[S11] Zhou QQ, Wang J, Tang W, Hu ZC, Xia ZY, Li XS, et al. Automatic detection and classification of rib fractures on thoracic CT using convolutional neural network: accuracy and feasibility. Korean journal of radiology. 2020;21(7):869.

[S12] Tajmir SH, Lee H, Shailam R, Gale HI, Nguyen JC, Westra SJ, et al. Artificial intelligence-assisted interpretation of bone age radiographs improves accuracy and decreases variability. Skeletal radiology. 2019;48(2):275–283.

[S13] Sim Y, Chung MJ, Kotter E, Yune S, Kim M, Do S, et al. Deep convolutional neural network–based software improves radiologist detection of malignant lung nodules on chest radiographs. Radiology. 2020;294(1):199–209.

[S14] Lee JH, Ha EJ, Kim D, Jung YJ, Heo S, Jang YH, et al. Application of deep learning to the diagnosis of cervical lymph node metastasis from thyroid cancer with CT: external validation and clinical utility for resident training. Eur Radiol. 2020;p. 3066–3072.

[S15] Kozuka T, Matsukubo Y, Kadoba T, Oda T, Suzuki A, Hyodo T, et al. Efficiency of a computer-aided diagnosis (CAD) system with deep learning in detection of pulmonary nodules on 1-mm-thick images of computed tomography. Japanese Journal of Radiology. 2020;38(11):1052–1061.

[S16] Jang S, Song H, Shin YJ, Kim J, Kim J, Lee KW, et al. Deep learning–based automatic detection algorithm for reducing overlooked lung cancers on chest radiographs. Radiology. 2020;296(3):652–661.

[S17] Cha KH, Hadjiiski LM, Cohan RH, Chan HP, Caoili EM, Davenport MS, et al. Diagnostic accuracy of CT for prediction of bladder cancer treatment response with and without computerized decision support. Academic radiology. 2019;26(9):1137–1145.

[S18] Cai SL, Li B, Tan WM, Niu XJ, Yu HH, Yao LQ, et al. Using a deep learning system in endoscopy for screening of early esophageal squamous cell carcinoma (with video). Gastrointestinal endoscopy. 2019;90(5):745–753.

[S19] Sato Y, Takegami Y, Asamoto T, Ono Y, Hidetoshi T, Goto R, et al. Artificial intelligence improves the accuracy of residents in the diagnosis

of hip fractures: a multicenter study. BMC Musculoskeletal Disorders. 2021;22(1):1–10.

[S20] Yu Q, Huang K, Zhu Y, Chen X, Meng W. Preliminary results of computer-aided diagnosis for magnetic resonance imaging of solid breast lesions. Breast cancer research and treatment. 2019;177(2):419–426.

[S21] Choi SY, Park S, Kim M, Park J, Choi YR, Jin KN. Evaluation of a deep learning-based computer-aided detection algorithm on chest radiographs: Case–control study. Medicine. 2021;100(16).

[S22] Choi JW, Cho YJ, Ha JY, Lee YY, Koh SY, Seo JY, et al. Deep learning-assisted diagnosis of pediatric skull fractures on plain radiographs. Korean Journal of Radiology. 2022;23(3):343.

[S23] Shang S, Huang C, Yan W, Chen R, Cao J, Zhang Y, et al. Performance of a computer aided diagnosis system for SARS-CoV-2 pneumonia based on ultrasound images. European Journal of Radiology. 2022;146:110066.

[S24] Roller R, Mayrdorfer M, Duettmann W, Naik MG, Schmidt D, Halleck F, et al. Evaluation of a clinical decision support system for detection of patients at risk after kidney transplantation. Frontiers in public health. 2022;10:979448. https://doi.org/10.3389/fpubh.2022.979448.

[S25] Wang X, Sun Z, Xue H, Qu T, Cheng S, Li J, et al. A deep learning algorithm to improve readers' interpretation and speed of pancreatic cystic lesions on dual-phase enhanced CT. Abdominal Radiology. 2022;47(6):2135–2147.

[S26] Yacoub B, Varga-Szemes A, Schoepf UJ, Kabakus IM, Baruah D, Burt JR, et al. Impact of artificial intelligence assistance on chest CT interpretation times: a prospective randomized study. American Journal of Roentgenology. 2022;219(5):743–751.

[S27] Wei Q, Zeng SE, Wang LP, Yan YJ, Wang T, Xu JW, et al. The added value of a computer-aided diagnosis system in differential diagnosis of breast lesions by radiologists with different experience. Journal of Ultrasound in Medicine. 2022;41(6):1355–1363.

[S28] Wataya T, Yanagawa M, Tsubamoto M, Sato T, Nishigaki D, Kita K, et al. Radiologists with and without deep learning–based computer-aided diagnosis: comparison of performance and interobserver agreement for characterizing and diagnosing pulmonary nodules/masses. European Radiology. 2023;33(1):348–359.

[S29] Toda N, Hashimoto M, Iwabuchi Y, Nagasaka M, Takeshita R, Yamada M, et al. Validation of deep learning-based computer-aided detection

software use for interpretation of pulmonary abnormalities on chest radiographs and examination of factors that influence readers' performance and final diagnosis. Japanese Journal of Radiology. 2022;p. 1–7.
